# Black esophagus in forensic autopsies: impressive finding and cause of death?

**DOI:** 10.1007/s12024-025-01048-x

**Published:** 2025-07-24

**Authors:** Bianca Beltrame, Stefan Pittner, Thomas Keller, Fabio C. Monticelli

**Affiliations:** 1 Department of Forensic Medicine, University of Brescia, 25123 Brescia, Italy; 2 Department of Forensic Medicine and Forensic Psychiatry, Paris- Lodron University of Salzburg, Salzburg, 5020 Austria

**Keywords:** Black esophagus, Autopsy, Pneumonia, Chronic alcohol abuse, Sudden death

## Abstract

Black esophagus (BE) is characterized by a discoloration of the esophageal mucosa, commonly arising from acute esophageal necrosis. The underlying pathogenesis of BE is poorly understood though it is frequently associated with comorbidities, such as diabetes mellitus, alcohol abuse, infections. Determining the cause of death in cases involving BE at autopsy can be particularly challenging. The report presents the case of a 45-year-old man with a history of alcohol abuse. Autopsy revealed extensive BE along with bilateral pneumonia. Cause of death was determined to be severe pneumonia in combination with acute esophageal necrosis, against a background of chronic alcohol abuse. This case underscores the importance of a thorough forensic investigation, including anamnestic information, autopsy findings and histopathological examination, in order to accurately establish the cause of death, even in presence of dramatic findings such as BE.

## Introduction

The term “black esophagus” (BE) refers to cases in which the esophageal mucosa exhibits a black discoloration which typically ends abruptly at the gastroesophageal junction [1]. The etiology of BE is multifactorial, with the majority of cases – particularly when observed during autopsy – resulting from acute esophageal necrosis. However, differential diagnoses should also be considered, including malignant tumors, acanthosis nigricans of the esophagus [2] or ingestion of acid substances [3]. Specifically, the ingestion of sodium nitrate, either accidental or intentional (for suicidal purposes [4]), induces toxicity by oxidizing ferrous (Fe²⁺) hemoglobin to ferric (Fe³⁺) hemoglobin, resulting in the formation of methemoglobin, thereby leading to cellular hypoxia and cyanosis. Sodium nitrate can be detected through toxicological analysis of gastric contents using a specific spectrophotometric methodology based on the Griess reagent for sodium nitrite; however, the presence of nitrates has also been reported in stomach contents, urine, pericardial fluid and cerebrospinal fluid [5, 6]. Similarly, the ingestion of citric acid can induce BE, resulting in death when combined with inhibition of blood coagulation due to a reduction in the concentration of calcium ions in the blood, since citric acid has a chelating effect on calcium ions [3].

To address this diagnostic challenge, histological examination has proven effective in detecting full-thickness necrosis of the esophageal wall. This necrosis may be demarcated by a broad zone of neutrophilic granulocytes located in the upper submucosa [7], thus supporting an ischemic origin.

Despite this surprising autopsy finding, the role of the BE in determining death remains a topic of debate in the literature. This is attributable to its rare occurrence and the fact that its underlying pathogenesis is not yet fully understood. In the majority of cases observed during endoscopy, BE resolves spontaneously [8].

The prevalence of BE is difficult to establish. When identified during endoscopic examinations, the prevalence of BE in recent studies ranges from 0.06% (16 cases of BE out of 25,970 upper gastrointestinal endoscopies) [9] to 0.28% (29 cases of BE among 10,000 examinations conducted over five years) [10].

In the context of autopsies, establishing the prevalence of BE is even more challenging, as it must be interpreted in relation to clinical data to determine whether it is an incidental finding or a cause of death. A systematic review by Schizas et al. [11]. analyzed 114 patients with BE discovered during hospitalization, reporting that 32 patients (29.9%) died either during the initial hospital stay or subsequently at follow-up. Nevertheless, the study does not report the presence of comorbidities (either their number or nature) in the patients who died; therefore, mortality cannot be attributed exclusively to BE, as other conditions may be responsible. Furthermore, a frequently cited study on the prevalence of BE in autopsy findings dates back to 1974 [12], predating the introduction of the term “black esophagus” by Goldenberg in 1990 [13].

Moreover, most autopsy cases document at least one comorbidity along a BE, typically sufficient explanations for fatality. These include diabetes mellitus [14–16], alcohol abuse [17], and infections [13].

Two case reports [7, 18] in which death was attributed to BE in combination with duodenal ulcers and fatal cardiac arrhythmias secondary to cellular ischemia, respectively, have been questioned [19, 20]. In the first case, it was suggested that the BE was “*only a clue to another catastrophic event that went unnoticed such as a cardiac arrhythmia*”. In the second case, it was suggested that the finding of BE on postmortem examination “*should always be considered a potential clue to sometimes concealed primary event*”.

The following report details the case of a 45-years-old man, who was discovered dead in his residence. His medical records included epilepsy with recurrent seizures and chronic alcohol abuse. At autopsy, a extensive BE was identified, however all available information – from medical history to histological investigations – had to be considered. Ultimately, the cause of death was determined to be pneumonia together with an acute esophageal necrosis (black esophagus).

### Case report

A 45-year-old man was found dead in his apartment in the living room, where multiple bloodstains were observed on and near the body. An autopsy was performed to determine the cause of death and to assess the possible involvement of third parties.

From medical records, epilepsy with recurrent seizures was diagnosed, with a prescription of levetiracetam as a treatment. The individual was known as a chronic alcohol abuser. Alcohol dependence of at least four years’ duration, associated with signs of toxic liver disease, was documented. His mother – the last person with whom he spoke on the phone a few hours prior to death – reported that he appeared to be in an altered state.

Furthermore, approximately eighteen months prior, he sustained a subdural hematoma following an accidental fall, after which neurological disturbances were noted.

**Fig. 1:**
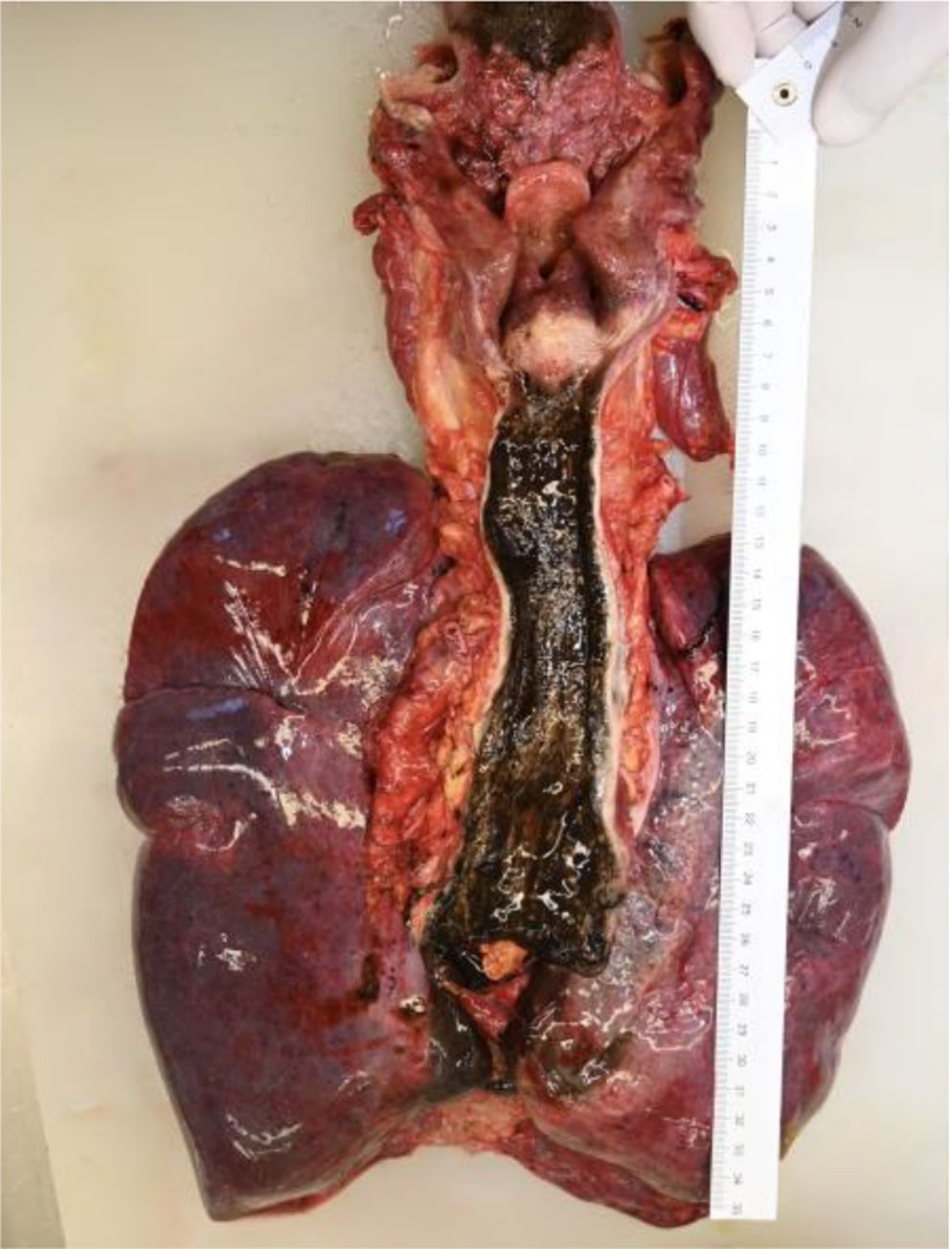
dark green- black esophageal mucosa, with a sharp demarcation at the gastroesophageal junction

External examination revealed several recent contused lacerations and abrasions on the face and both upper and lower limbs, accompanied by abundant blood smearing. At autopsy, the esophageal mucosa ranged in color from dark green to black (Fig. 1), with a sharp demarcation at the gastroesophageal junction. Beyond this point, the gastric mucosa showed a beige-gray appearance. The trachea and airways contained copious mucus and the lungs displayed signs of pulmonary edema. The liver was soft in consistency and light yellow in color. The duodenum and small intestine contained green-black material, which progressively darkened to black-red in the distal bowel, culminating in black feces in the colon. Fractures of the left 10th and 11th ribs were identified in the posterior section of the rib cage.

No pathological findings were observed in the brain or in the heart.

**Fig. 2:**
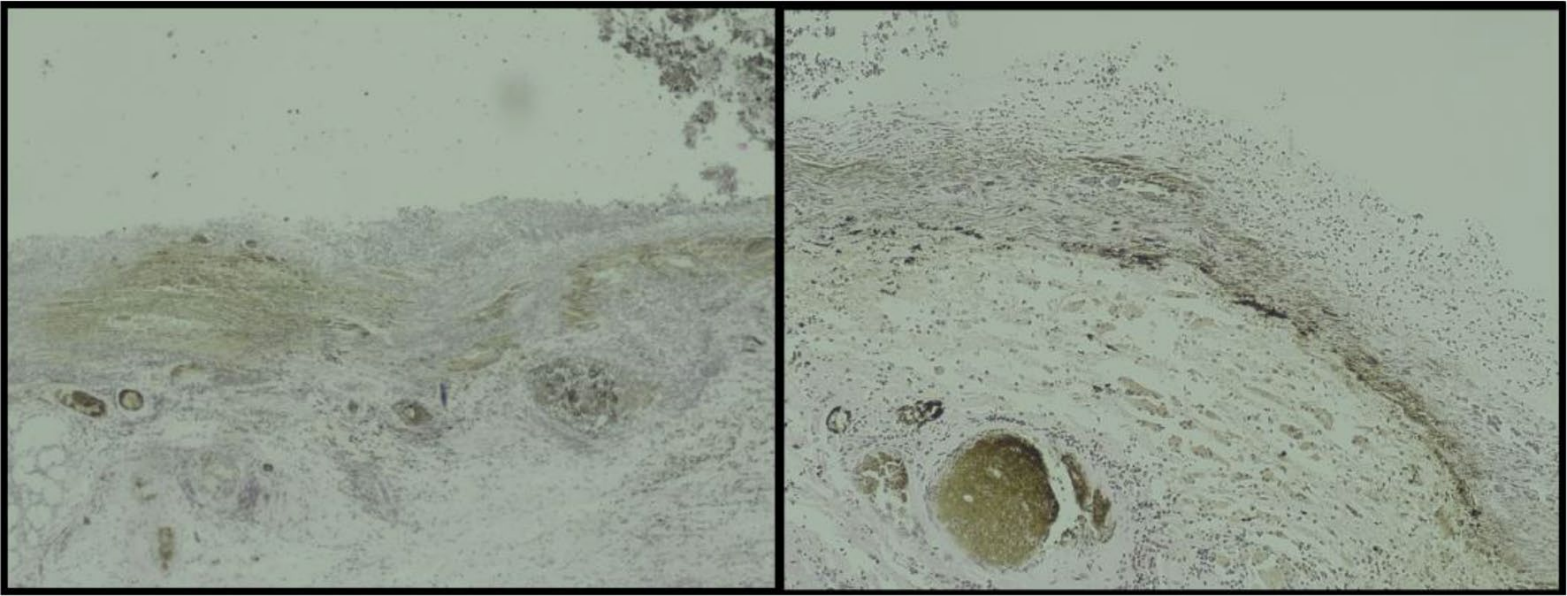
Acute necrosis of the esophageal mucosa. Hematoxylin and eosin, 4 × 0.10 (left) and 10 × 0.25 (right)

**Fig. 3:**
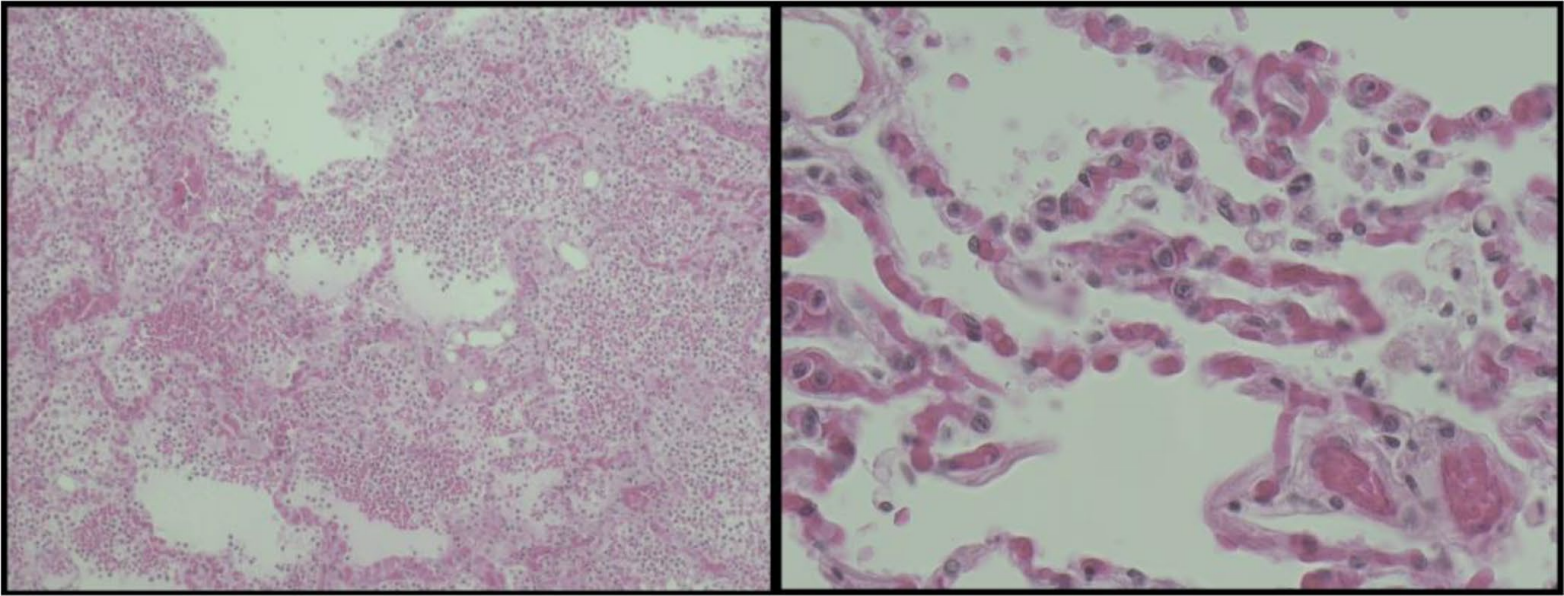
Lung parenchyma with massive lymphocytic infiltration - pneumonia. Hematoxylin and eosin, 10 × 0.25 (left) and 40 × 0.75 (right)

Histological sections stained with hematoxylin and eosin revealed acute necrosis of the esophageal mucosa (Fig. 2), with ulceration and a clear transition to the gastric mucosa, in which no neoplastic cells were identified. The lung parenchyma showed mild emphysema and severe acute pneumonia (Fig. 3). Hepatic histology confirmed macrovesicular steatosis, involving at least 75% of the parenchyma.\.

Toxicological analyses for alcohol concentration performed in femoral blood, urine and cerebrospinal fluid gave as a results 0.67%_o_, 0.97%_o_ and 0.87%_o_, respectively. All other forensic toxicological analyses for drugs and centrally acting substances were negative.

The diffuse bruises and posterior fractures of the ribs were attributed to accidental falls occurring prior to death.

The cause of death was determined to be severe pneumonia in combination with acute esophageal necrosis, in a individual with a history of chronic alcohol abuse and underlying liver disease. The manner of death was ultimately classified as natural, thereby excluding any third-party involvement.

## Discussion

In this case, the cause of death was identified as a combination of pulmonary pneumonia and the acute esophageal necrosis (resulting in a BE). Although BE was a dramatic autopsy finding and the deceased was known to be a chronic alcohol abuser, this alone was insufficient to account for death. Pneumonia was suspected at autopsy and subsequently confirmed by histological examinations.

The occurrence of BE in individuals with a history of alcohol abuse [21] or recent alcohol consumption [22] remains a topic of debate in the literature. Na [23] proposes several mechanisms of death in the case of acute necrotizing esophagitis of a heavy drinker including inflammation from the severe necrosis in the distal esophagus, hypovolemic shock due to gastrointestinal hemorrhage (in this case melena and/or hematemesis should be the clinical presentation) or eventually due to the aggravation of the underlying diseases, such as liver cirrhosis, diabetes, hypertension, and chronic renal insufficiency, sepsis and immunosuppression.

Despite these proposed mechanisms, in many cases the cause of death remains undetermined. In the case described by Unuma [24], autopsy revealed the presence of BE, a modest concentration of alcohol in both blood and urine, leading to the authors suggestion “*that the direct cause might be attributed to cardiac arrhythmias triggered by a Valsalva maneuver during vomiting*,* or to electrolyte imbalance resulting from swallowing difficulties and an eating disorder*,* as inflammation had extended to the deep mucosal layers*” [24].

However, even in the case of a young man with acute esophageal necrosis associated with alcoholic ketoacidosis after excessive alcohol consumption, prolonged starvation and self-reported increased intake of venlafaxine and quetiapine, reported by Egli et al. [22], a full recovery was achieved with conservative medical management (i.e. no surgery but controlled fluid resuscitation).

Older age thus seems to be a relevant factor when associated with BE and alcohol consumption among patients reported in various studies [19, 21] – and the consequent presence of comorbidities. Anyway, in the present case the association between BE and alcohol consumption does not appear entirely satisfactory in explaining sudden death, particularly giving the victim’s young age.

A different consideration applies, however, to the association between BE and various infections. Although such correlations are rarely reported in the literature, some cases report associations with respiratory infection [15], septicemia [13], cytomegalovirus (CMV) infections [25]. Notably, complete recovery was documented in some instances. For example, Trappe [26] described a case of BE associated with CMV infection in which the patient fully recovered. Similarly, Alkhalil [27] reported a full recovery in a patient suffering from BE in the context of urosepsis and diabetic ketoacidosis due to uncontrolled type 2 diabetes mellitus.

A key difference between fatal and non-fatal cases appears to be the age of the patients. Rodriguez Fernandez [15] reports a 81-years-old man with BE, diabetes mellitus type 2, chronic alcoholism and respiratory infection, who died five days after BE was diagnosed. Monteiro [13] described a 75-year-old woman who succumbed to multiple organ failure from septicemia. In contrast, the patients in the non-fatal cases were significantly younger – 54-years-old in Trappe’s case [26] and 60 years old the report from Alkhalil [27].

In the present case, the decedent was 45-year-old man with a documented history of chronic alcohol abuse. The most striking macroscopic finding at autopsy was a extensively discolored, necrotic esophagus, consistent with BE. Histological examinations further revealed diffuse pneumonia, consistent with the abundant mucus observed in the trachea and lungs.

Taking into account the literature and the autopsy findings, the chronic alcohol intoxication likely contributed to death through indirect mechanisms, through hepatotoxic liver disease associated with coagulopathy and impaired mobility, as suggested by signs of recent falls.

In determining the cause of death, the two key findings were considered: the presence of BE in an individual with chronic alcohol abuse, and the histologically confirmed pneumonia. Ultimately, the cause of death was attributed to severe pneumonia in combination with acute esophageal necrosis/BE, in a patient with chronic alcohol abuse and underlying liver disease.

This case highlights the importance of critically assessing the clinical history and carefully weighing the significance of each finding. While a massive black esophagus in a patient with chronic alcohol abuse might suggest an obvious diagnostic conclusion, such an approach would have been inadequate from an anatomopathological perspective, particularly given our still incomplete understanding of the underlying pathological mechanisms of BE.

## Conclusion

In conclusion, this case underscores the necessity of comprehensively evaluating all available findings to accurately assess their significance in the specific context. Although BE is a striking finding at autopsy that potentially leads to a premature conclusion – current knowledge about the pathophysiological mechanisms underlying BE remains limited, particularly in cases of sudden death.

The pathological mechanisms that lead to black esophagus pigmentation following a necrotic process should be studied, in order to identify possible therapeutic strategies and also those factors that favor a negative outcome. Considering the rarity of BE during endoscopic exams and its frequent resolution without specific treatment, forensic pathologists must avoid over interpretation of its presence in isolation. Instead, it is essential to contextualize BE within the clinical and pathological picture and take into account – and often integrate – common comorbidities associated with the condition, such as diabetes mellitus, chronic alcohol abuse, and infections, when establishing the cause of death.

## Key points

1. Black esophagus (BE) is a black discoloration of the esophageal mucosa.
2. BE is frequently associated with diabetes mellitus, alcohol abuse, infections, etc.
3. Determining the cause of death in cases involving BE can be challenging.
4. To establish the cause of death, anamnestic information, autopsy findings and histopathological examination must be considered.
